# A surgical case of ciliated muconodular papillary tumor of the lung

**DOI:** 10.1186/s44215-022-00024-z

**Published:** 2023-03-30

**Authors:** Hiroaki Shidei, Hiroe Aoshima, Akira Ogihara, Tamami Isaka, Hiromi Onizuka, Yoji Nagashima, Yukio Nakatani, Masato Kanzaki

**Affiliations:** 1 Department of Thoracic Surgery, Tokyo Women’s Medical University, Tokyo, Japan; 2 Department of Surgical Pathology, Tokyo Women’s Medical University, Tokyo, Japan; 3 Department of Pathology, Yokosuka Kyosai Hospital, Yokosuka-shi, Kanagawa Japan

**Keywords:** Ciliated muconodular papillary tumor, Lung nodule, EGFR

## Abstract

Ciliated muconodular papillary tumor (CMPT) is a rare type of tumor with both benign and malignant characteristics. Herein, we report the surgical case of a 65-year-old man with CMPT. Chest computed tomography revealed a solitary cavitary lesion with a maximum diameter of 11-mm in S^10^b of the right lower lung. A thoracoscopic lung wedge resection was subsequently performed. On microscopic examination, the tumor was composed of highly columnar cells with tubular-to-papillary and cystic growth patterns. On immunostaining, it was positive for the epidermal growth factor receptor. The tumor was diagnosed as a CMPT, exhibiting no recurrence after two years of follow-up.

## Introduction

Ciliated muconodular papillary tumor (CMPT) is a rare lung tumor that was first reported in 2002. This tumor involves ciliated, goblet, and basal cell proliferation with mucin secretion [1]. On computed tomography (CT), CMPT commonly presents as ground-glass opaque peripheral nodules, while cavitary formation is rarely observed. This study presents a surgical case of CMPT in a 65-year-old man with a cavitary lesion in the right lower lung field identified on chest CT. The diagnostic pitfalls and considerations are further discussed.

### Case

**Fig. 1:**
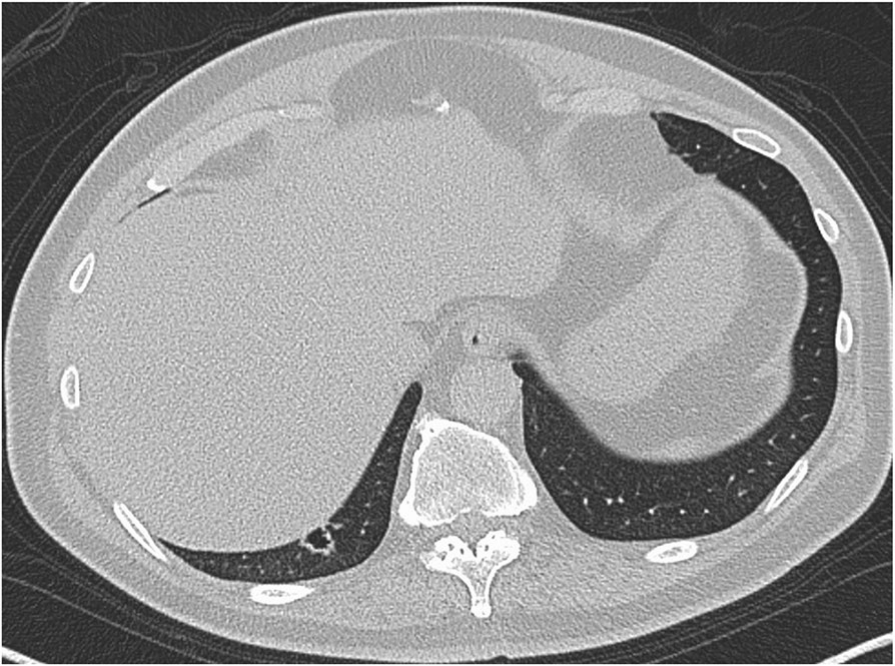
Chest computed tomography (CT) showing a cavitary lesion in S10b of the right lower lobe

**Fig. 2:**
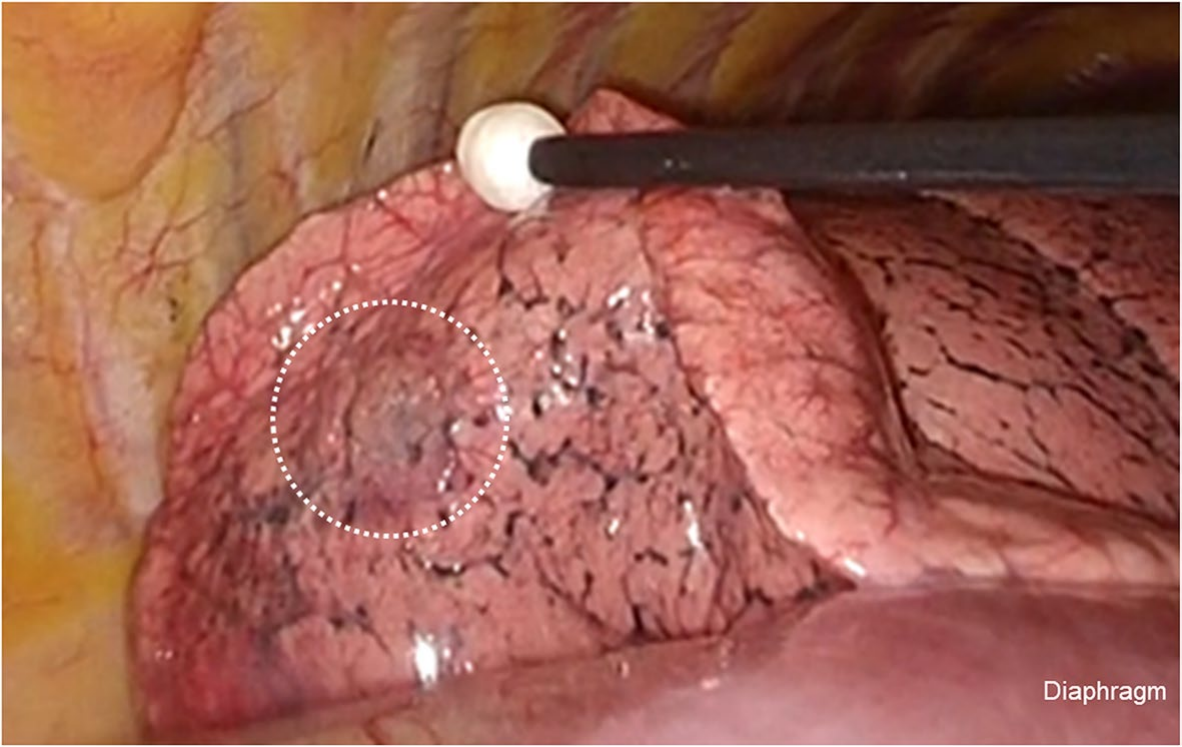
Intraoperative findings revealed the nodule (white dotted circle) in the dorsobasal segment of the right lung. The photograph was extracted from the digital video data

**Fig. 3:**
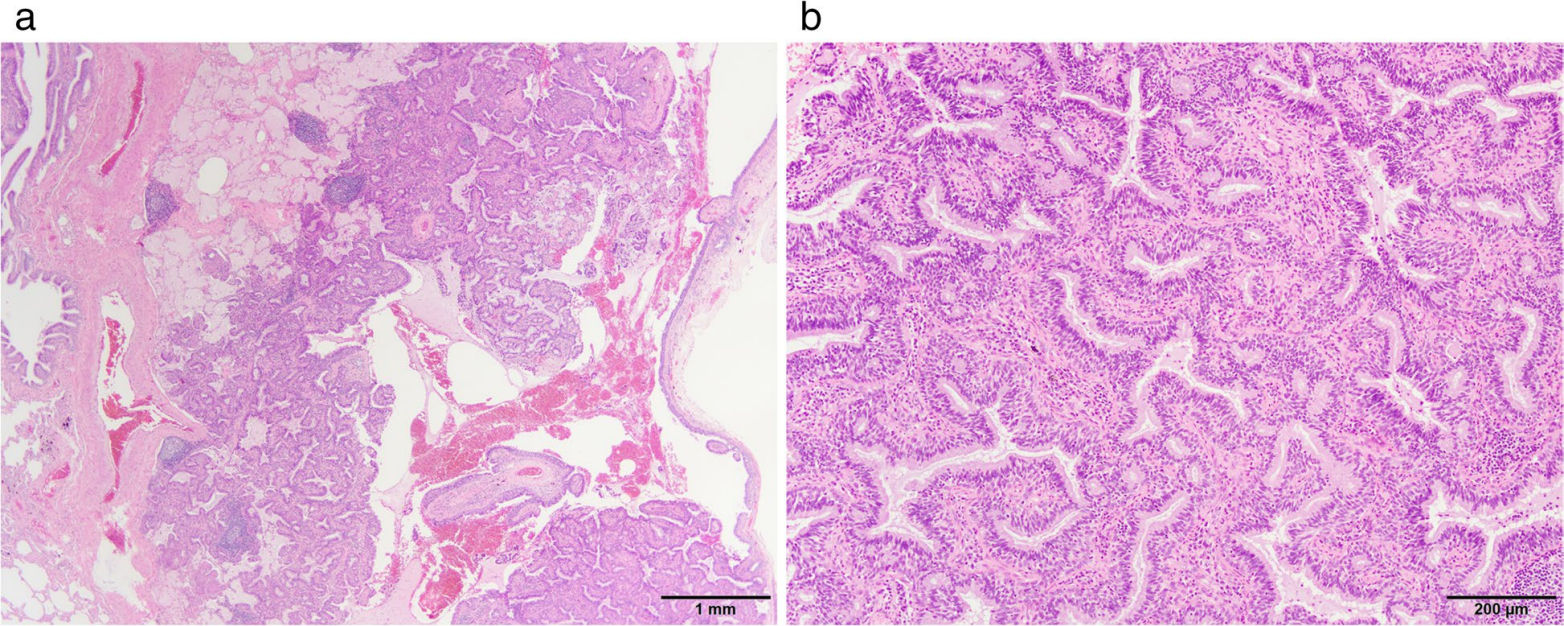
Hematoxylin and eosin staining showed that the tumor was composed of tall columnar cells exhibiting a tubular-to-papillary and cystic growth pattern. **A** Low-power field. **B** High-power field

**Fig. 4:**
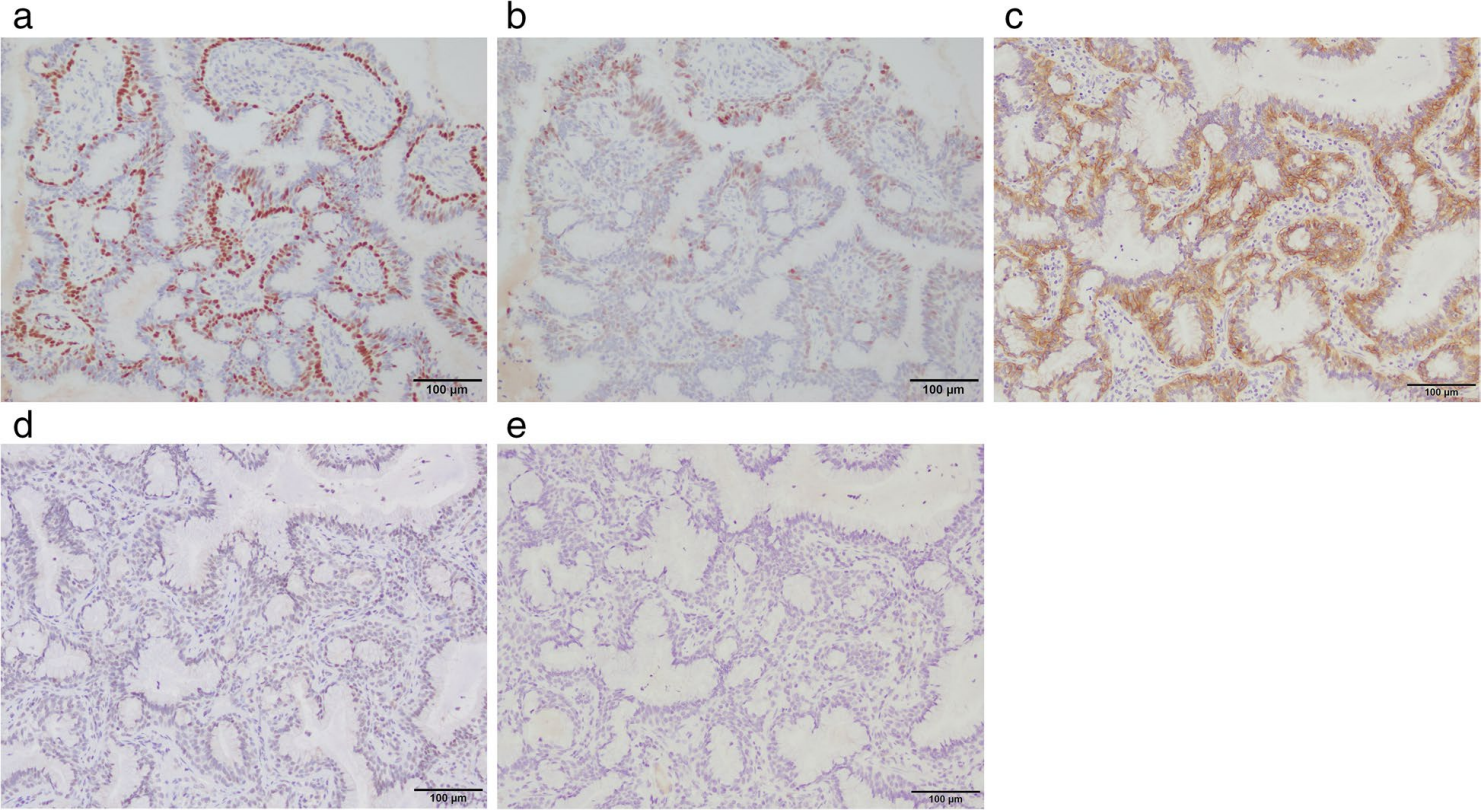
Immunohistochemistry. **a** Basal cells were positive for p40. **b** Alveolar epithelial cells were positive for TTF-1. **c** Tall columnar cells strongly expressed EGFR. Conversely, **d** BRAF V600E and **e** ALK expression was not observed

A 65-year-old man had been referred to our hospital for evaluation 4 years prior, after the detection of a cavitary lesion on chest CT during routine health screening. He had a history of hyperlipidemia and impaired glucose tolerance. Laboratory examination revealed elevated triglyceride and LDL-cholesterol levels. The serum levels of the tumor markers carcinoembryonic antigen (CEA), pro-gastrin-releasing peptide, and squamous cell carcinoma antigen were within the normal limits. CT revealed a solitary cavitary lesion with a maximum diameter of 11 mm in S^10^b of the right lower lung (Fig. 1). ^18^F-fluorodeoxyglucose (FDG) positron emission tomography (PET) revealed no abnormal FDG accumulation in the tumor or lymph nodes. Thoracoscopic lung wedge resection was subsequently performed under one-lung anesthesia to verify the presence of a primary malignancy (Fig. 2). The resected specimen showed an 11-mm cavity, and intraoperative analysis of the frozen section revealed only inflammatory changes; therefore, additional lobectomy was not performed. On microscopic examination, the tumor was composed of tall columnar cells, exhibiting tubular-to-papillary and cystic growth patterns (Fig. 3a, b). Lymphocyte infiltration was further observed in the interstitium. On immunohistochemical staining, basal cells were positive for p40, and alveolar epithelial cells were positive for thyroid transcription factor 1 (TTF-1) (Fig. 4a, b). Tall columnar cells strongly expressed epidermal growth factor receptor (EGFR) and v-raf murine sarcoma viral oncogene homolog B1 V600E (BRAF V600E) and anaplastic lymphoma kinase (ALK) were not expressed (Fig. 4c–e). The final pathological diagnosis was CMPT. The postoperative course was uneventful, with no recurrence 2 years later.

## Discussion

CMPT (which was first reported in 2002) is a rare tumor that develops in the peripheral lung. This lesion exhibits papillary growth during alveolar replacement, and ciliated and goblet cells are accompanied by mucus production [1]. CMPT was classified as an adenoma in the 5th edition of the WHO classification [2]; however, CMPT has been described as having both benign and malignant features. The presence of cilia, basal cells, and a low Ki-67 index are all indicators of a benign tumor. Conversely, findings, suggestive of a malignant tumor include a ruptured alveolar structure, central fibrosis, proliferation along the alveolar walls and skip lesions, lack of encapsulation, micropapillary patterns, and positive staining for CEA. The immunohistochemical findings of this tumor are similar to those of lung adenocarcinoma. CMPT is typically positive for CEA, TTF-1, and cytokeratin (CK) 7, but negative for CK20 [3, 4]. Although the malignant potential of CMPT remains unknown, some studies have associated it with various gene alterations. Recently, EGFR, ALK, AKT1, KRAS, BRAF, and HRAS gene mutations were all detected via gene analyses among patients with CMPT [5–7]. These findings are suggestive of a neoplastic change. Thus, their malignant potential requires further investigation.

CMPT is frequently detected incidentally as a small, peripheral, ground-glass opacity or nodule on CT. In a previous study examining 38 cases of CMPTs (17 men and 21 women), the mean age at identification was 68 years (range 50–84 years), and the average tumor diameter was 1.1 cm (range 0.4–4.5 cm) [8]. Most CMPT lesions developed in the peripheral lesions of the lung with approximately 79.4–81.5% occurring in the lower lobe [8, 9]. CT findings included nodular shadows in 28 patients (73.7%) and hollow shadows and ground-glass-like shadows in the remaining 10 (26.3%). In another study, the CT findings of 16 patients with CMPTs, including eight (50.0%) with solid nodules, seven (43.8%) with high-density ground-glass nodules (GGNs), and one (6.3%) with pure GGNs, were examined. CMPTs grew very slowly at 0.49 mm/year in maximal diameter. Moreover, 10 out of 16 lesions (62.5%) were found near the pleura [9]. Based on this, it may be difficult to distinguish CMPT from lung cancer, when pleural changes are present.

FDG-PET findings of 15 patients with surgically resected CMPTs showed moderate FDG uptake in one patient, with the maximum standardized uptake values (SUVmax) of 3.67. The remaining 14 patients showed mild FDG uptake, with the SUVmax ranging from 0.57 to 1.35. According to the relationship between FDG accumulation and pathological lymphocyte infiltration, cases with mild FDG accumulation exhibit minimal lymphocyte infiltration and large amounts of mucin. Meanwhile, cases with moderate FDG accumulation exhibit significant lymphatic infiltration [10].

Since CMPT often presents as a small nodule in the peripheral lesion of the lung, it is difficult to diagnose on bronchoscopy. As such, surgical resection is often performed to confirm the diagnosis. In 38 CMPT cases, partial resection was performed in 27 (71.1%), while segmentectomy was performed in one, and lobectomy was performed in 10 [8]. When sufficient excision margins are obtained in the excision range, a partial excision is considered curative [11]. In this case, the maximum tumor diameter was 11 mm, and malignant findings were not observed in the intraoperative analysis of the frozen sections. Therefore, a sufficient resection margin was secured, and the operation was successfully completed via partial resection.

The prognosis for CMPT is good, and no recurrence or metastasis was reported during the mean follow-up period of 33.8 months [8]. Since the pathological findings of CMPT exhibit both benign and malignant characteristics, careful follow-up is necessary. In conclusion, this study reports a rare case of CMPT, which was managed by thoracoscopic lung wedge resection, with no recurrence detected in 2 years of follow-up.

## Data Availability

Not applicable.

## Abbreviations

CMPT: Ciliated muconodular papillary tumor
CT: Computed tomography
CEA: Carcinoembryonic antigen
FDG: 18 F-fluorodeoxyglucose
PET: Positron emission tomography
EGFR: Epidermal growth factor receptor
ALK: Anaplastic lymphoma kinase
CK: Cytokeratin
AKT1: Serine/threonine kinase 1
KRAS: v-Ki-ras2 Kirsten rat sarcoma viral oncogene homolog
BRAF: v-raf murine sarcoma viral oncogene homolog B1
SUVmax: Maximum standardized uptake values
